# Chemosensitivity-directed therapy compared to dacarbazine in chemo-naive advanced metastatic melanoma: a multicenter randomized phase-3 DeCOG trial

**DOI:** 10.18632/oncotarget.18635

**Published:** 2017-06-27

**Authors:** Selma Ugurel, Carmen Loquai, Patrick Terheyden, Dirk Schadendorf, Erika Richtig, Jochen Utikal, Ralf Gutzmer, Knuth Rass, Cord Sunderkötter, Annette Stein, Michael Fluck, Martin Kaatz, Uwe Trefzer, Katharina Kähler, Rudolf Stadler, Carola Berking, Christoph Höller, Laura Kerschke, Lutz Edler, Annette Kopp-Schneider, Jürgen C. Becker

**Affiliations:** ^1^ Department of Dermatology, University Hospital of Essen, Essen, Germany; ^2^ Department of Dermatology, University Hospital of Würzburg, Würzburg, Germany; ^3^ Department of Dermatology, University Hospital of Mainz, Mainz, Germany; ^4^ Department of Dermatology, University Hospital of Lübeck, Lübeck, Germany; ^5^ Translational Skin Cancer Research, Deutsches Konsortium für Translationale Krebsforschung (DKTK), Essen, Germany; ^6^ Department of Dermatology, Medical University of Graz, Graz, Austria; ^7^ Skin Cancer Unit, German Cancer Research Center (DKFZ), Heidelberg, Germany; ^8^ Department of Dermatology, Venereology and Allergology, University Medical Center Mannheim, Ruprecht-Karl University of Heidelberg, Mannheim, Germany; ^9^ Department of Dermatology and Allergy, Skin Cancer Center Hannover, Hannover Medical School, Hannover, Germany; ^10^ Department of Dermatology, The Saarland University Hospital, Homburg/Saar, Germany; ^11^ Department of Dermatology, University Hospital of Münster, Münster, Germany; ^12^ Department of Dermatology, University Hospital of Dresden, Dresden, Germany; ^13^ Department of Internal Medicine, Fachklinik Hornheide, Hornheide, Germany; ^14^ Department of Dermatology, University Hospital of Jena, Jena, Germany; ^15^ Department of Dermatology, University Hospital Charite, Berlin, Germany; ^16^ Department of Dermatology, University Hospital of Kiel, Kiel, Germany; ^17^ Department of Dermatology, Johannes Wesling Klinikum, Minden, Germany; ^18^ Department of Dermatology, University Hospital of Munich, Munich, Germany; ^19^ Department of Dermatology, Medical University of Vienna, Vienna, Austria; ^20^ Division of Biostatistics, German Cancer Research Center, Heidelberg, Germany; ^21^ Institute of Biostatistics and Clinical Research, University of Münster, Münster, Germany

**Keywords:** melanoma, chemosensitivity, individualized chemotherapy, phase-3 trial

## Abstract

Chemotherapy still plays an important role in metastatic melanoma, particularly for patients who are not suitable or have no access to highly efficacious new therapies. Pre-therapeutic chemosensitivity testing might be useful to identify optimal chemotherapy regimens for individual patients. This multicenter randomized phase-3 trial was aimed to test for superiority of chemosensitivity-directed combination chemotherapy compared to standard dacarbazine monochemotherapy, and to demonstrate the chemosensitivity test result as prognostic in metastatic melanoma. Chemo-naive patients with advanced melanoma were biopsied from metastatic lesions. Tumor cells were isolated and tested *ex-vivo* for sensitivity to chemotherapeutic agents using an ATP-based viability assay. Patients with evaluable test results were randomly assigned to receive either chemosensitivity-directed combination chemotherapy (paclitaxel+cisplatin, treosulfan+gemcitabine, treosulfan+cytarabine), or dacarbazine. The primary study endpoint was overall survival (OS). After inclusion of 287 patients and a median follow-up of 26 months, the per-protocol population (n=244) showed no difference in OS between chemosensitivity-directed therapy and dacarbazine (median 9.2 vs 9.0 months, HR=1.08, p=0.64). The disease control rate (CR+PR+SD) tended to be higher in patients treated with chemosensitivity-directed therapy (32.8% vs 23.0%, p=0.088); objective response rates (CR+PR) showed no difference between groups (10.7% vs 12.3%, p=0.90). Patients whose tumors were tested chemosensitive showed no better OS or response rate than patients with chemoresistant tumors. Severe toxicities (CTC grade 3-4) were significantly more frequently observed with chemosensitivity-directed combination chemotherapy than with dacarbazine (40.2% vs 12.3%, p<0.0001). These results indicate, that chemosensitivity-directed combination chemotherapy is not superior to dacarbazine, but leads to significantly more severe toxicities.

## INTRODUCTION

Until recently, chemotherapy with dacarbazine (DTIC) served as the therapeutic standard in patients with inoperable metastatic melanoma, rendering response rates of about 10% and a median overall survival of 8 to 12 months [1, 2]. However, since 2010 this situation has changed drastically, with completely new strategies for the treatment of advanced melanoma demonstrating a profound improvement in patient outcome. These therapeutics include kinase inhibitors interacting with the BRAF/MEK signaling pathway, and immune checkpoint inhibitors targeting CTLA-4 or PD-1/PD-L1 [3–5]. These agents, either as monotherapies or combinations lead to a significantly improved prognosis with median overall survival times ranging from 15 to more than 25 months, and thus rapidly replaced chemotherapy as the main standard treatment of metastatic melanoma.

However, chemotherapy is still utilized in metastatic melanoma, mainly in patients lacking a targetable mutation in the BRAF/MEK signaling pathway, or developing secondary resistance to BRAF/MEK inhibitors, and/or in patients not suitable or refractory to immune checkpoint inhibition. Additionally and most importantly, there currently are several countries without or with limited access to these new drugs. Consequently, a significant number of patients are still receiving chemotherapeutic regimens, either dacarbazine-based monotherapy or different regimens of combination chemotherapy, in any therapy line during their course of disease. A current analysis of a real-world database from the USA reflecting the systemic therapy of metastatic melanoma patients since the introduction of the above mentioned new agents revealed, that of 1043 included patients 7% were treated with dacarbazine and 19% with the dacarbazine-based agent temozolomide [6]. Recent clinical trials in metastatic melanoma reported median PFS of 2-3 months and median OS of 9-11 months in chemotherapy comparator arms [7–11].

Thus, it still is an important issue to identify patients who are likely to benefit from chemotherapy, or even to define the best suitable chemotherapy regimen for an individual patient. Valid predictive markers of the outcome of anti-melanoma chemotherapy are currently not availabe. To adress this notion, an *ex-vivo* performed pre-therapeutic chemosensitivity test on living tumor cells obtained from the individual patient has been established [12]. Moreover, this test assay was already successfully tested in a phase-2 study in metastatic melanoma performed by the Dermatologic Cooperative Oncology Group (DeCOG) [13]. The study results demonstrated a significant correlation between the chemosensitivity test result and the response to chemotherapy. Also, patients whose tumors were tested as chemosensitive by definition of the test assay showed an improved survival as compared to patients whose tumors were tested as chemoresistant.

The present study was designed to demonstrate both, a prognostic value of the *ex-vivo* chemosensitivity test result, as well as a superiority of an individualized sensitivity-directed combination chemotherapy against the standard regimen dacarbazine monochemotherapy in chemo-naïve metastatic melanoma.

## RESULTS

### Patient characteristics and study flow

**Figure 1:**
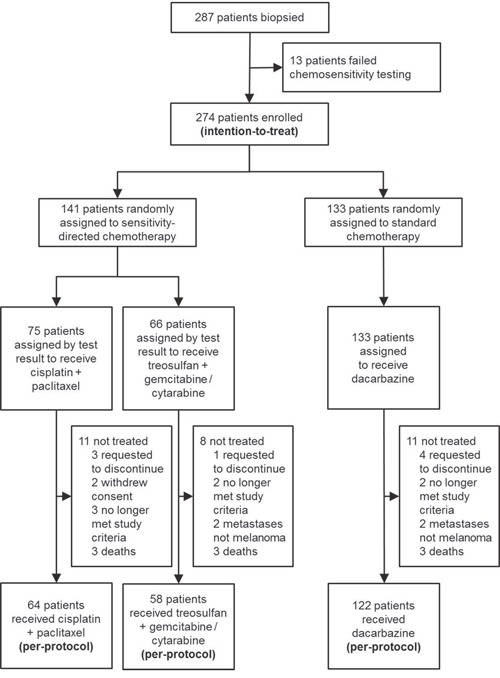
Schematic presentation of the study flow

Starting enrollment in November 2008, the recruitment rate decreased significantly during 2011 due to the introduction of BRAF/MEK pathway inhibitors and immune checkpoint blockers for the treatment of advanced metastatic melanoma. Thus, it was decided to stop the trial in October 2012 before the intended number of 360 patients could be enrolled. Between November 2008 and October 2012, 35 participating centers (see Acknowledgements) registered 287 patients, for whom tumor tissue biopsies were obtained and subjected to *ex-vivo* chemosensitivity testing. In 13 patients (4.5%) chemosensitivity testing failed due to low quantity or low viability of the extracted tumor cells. For the remaining 274 patients (intention-to-treat, ITT) evaluable test results were obtained, and the patients were subsequently randomized into the respective treatment arms, i.e. chemosensitivity-directed combination chemotherapy and dacarbazine monochemotherapy. Patient and tumor characteristics were balanced between both arms (Table 1). 30 patients did not receive study treatment after randomization for different reasons (see Figure 1); consequently, 244 patients were evaluable for all study endpoints (per-protocol, PP). Details on patient characteristics and study flow are presented in Table 1 and Figure 1.

**Table 1:** Patient characteristics at study enrollment

	ITT (n=274)			PP (n=244)		
	Standard chemotherapy (n=133)	Sensitivity-directed chemotherapy (n=141)		Standard chemotherapy (n=122)	Sensitivity-directed chemotherapy (n=122)	
	DTIC (n=133)	Treo + Gem/AraC (n=66)	Cis + Tax (n=75)	DTIC (n=122)	Treo + Gem/AraC (n=58)	Cis + Tax (n=64)
**Age**						
≤ 65	81 (60.9%)	45 (68.2%)	43 (57.3%)	74 (60.7%)	38 (65.5%)	37 (57.8%)
> 65	52 (39.1%)	21 (31.8%)	32 (42.7%)	48 (39.3%)	20 (34.5%)	27 (42.2%)
**Sex**						
Male	75 (56.4%)	39 (59.1%)	45 (60.0%)	70 (57.4%)	33 (56.9%)	40 (62.5%)
Female	58 (43.6%)	27 (40.9%)	30 (40.0%)	52 (42.6%)	25 (43.1%)	24 (37.5%)
**Localization of primary**						
Skin	102 (76.7%)	52 (78.8%)	60 (80.0%)	95 (77.9%)	47 (81.0%)	50 (78.1%)
Occult/MUP	18 (13.5%)	8 (12.1%)	10 (13.3%)	18 (14.8%)	6 (10.3%)	10 (15.7%)
Mucosa	6 (4.5%)	4 (6.1%)	3 (4.0%)	6 (4.9%)	4 (6.9%)	2 (3.1%)
Not specified	7 (5.3%)	2 (3.0%)	2 (2.7%)	3 (2.5%)	1 (1.7%)	2 (3.1%)
**AJCC M category**						
M1a	20 (15.0%)	13 (19.7%)	12 (16.0%)	20 (16.4%)	13 (22.4%)	11 (17.2%)
M1b	36 (27.1%)	15 (22.7%)	25 (33.3%)	33 (27.0%)	14 (24.2%)	23 (35.9%)
M1c	76 (57.8%)	38 (57.6%)	37 (49.4%)	69 (56.6%)	31 (53.4%)	30 (46.9%)
Not specified	1 (<1%)	0 (0%)	1 (1.3%)	0 (0%)	0 (0%)	0 (0%)
**ECOG performance status**						
0	96 (72.8%)	48 (72.7%)	52 (69.3%)	91 (74.6%)	42 (72.4%)	46 (71.9%)
1	36 (27.1%)	18 (27.3%)	22 (29.3%)	31 (25.4%)	16 (27.6%)	17 (26.5%)
Not specified	1 (<1%)	0 (0%)	1 (1.3%)	0 (0%)	0 (0%)	1 (1.6%)
**Serum LDH^1^**						
Normal (≤ ULN)	66 (49.6%)	38 (57.6%)	29 (38.7%)	63 (51.6%)	33 (56.9%)	24 (37.5%)
Elevated (> ULN)	67 (50.4%)	28 (42.4%)	46 (61.3%)	59 (48.4%)	25 (43.1%)	40 (62.5%)
**Sum of longest diameters of target lesions**						
≤ 10 cm	89 (66.9%)	50 (75.7%)	60 (80.0%)	85 (69.7%)	44 (75.9%)	50 (78.1%)
> 10 cm	34 (25.6%)	14 (21.3%)	9 (12.0%)	33 (27.0%)	13 (22.4%)	8 (12.5%)
Not specified	10 (7.5%)	2 (3.0%)	6 (8.0%)	4 (3.3%)	1 (1.7%)	6 (9.4%)
**Previous systemic therapy in stage III**						
Yes	59 (44.4%)	31 (47.0%)	42 (56.0%)	55 (45.1%)	30 (51.7%)	35 (54.7%)
No	67 (50.4%)	33 (50.0%)	31 (41.3%)	64 (52.4%)	27 (46.6%)	27 (42.2%)
Not specified	7 (5.2%)	2 (3.0%)	2 (2.7%)	3 (2.5%)	1 (1.7%)	2 (3.1%)
**Previous systemic therapy in stage IV**						
Yes	20 (15.0%)	4 (6.0%)	12 (16.0%)	18 (14.8%)	4 (6.9%)	11 (17.2%)
No	106 (79.8%)	59 (89.4%)	61 (81.3%)	101 (82.7%)	52 (89.7%)	51 (79.7%)
Not specified	7 (5.2%)	3 (4.6%)	2 (2.7%)	3 (2.5%)	2 (3.4%)	2 (3.1%)
**Localization of biopsy for chemosensitivity testing**						
Skin	59 (44.4%)	29 (43.9%)	44 (58.7%)	58 (47.5%)	27 (46.6%)	35 (54.7%)
Lymph node	40 (30.0%)	22 (33.3%)	21 (28.0%)	37 (30.3%)	20 (34.4%)	19 (29.7%)
Organ	30 (22.6%)	14 (21.2%)	9 (12.0%)	23 (18.9%)	11 (19.0%)	9 (14.1%)
Not specified	4 (3.0%)	1 (1.5%)	1 (1.3%)	4 (3.3%)	0 (0%)	1 (1.6%)
**Chemosensitivity test result**						
Chemosensitive (BICSI≤100)	34 (25.6%)	32 (48.5%)	8 (10.7%)	31 (25.4%)	29 (50.0%)	8 (12.5%)
Chemoresistant (BICSI>100)	99 (74.4%)	34 (51.5%)	67 (89.3%)	91 (74.6%)	29 (50.0%)	56 (87.5%)

Percentages are given per column, representing the treatment groups. ^1^Serum lactate dehydrogenase (LDH) values were classified according to the upper limits of normal (ULN) of the respective study centers. Previous systemic therapies in stage III or IV were immunotherapies in most of cases (interferon-alpha, interleukin-2, vaccination). DTIC, dacarbazine; Treo, treosulfan; Gem, gemcitabine; AraC, cytarabine; Cis, cisplatine; Tax, paclitaxel; AJCC, American Joint Committee on Cancer; ECOG, Eastern Cooperative Oncology Group.

### Heterogenous chemosensitivity

Chemosensitivity testing was performed on tumor biopsies obtained from different metastatic sites, with skin metastases representing the largest group (48.2%), followed by lymph node metastases (30.3%) and organ metastases (19.3%) (see Table 1). No significant differences in *ex-vivo* chemosensitivity profiles or BICSI values were observed between different metastatic sites. Since one repetition of tumor biopsy and chemosensitivity testing was allowed in each patient, the rate of non-evaluable testing and exclusion from randomization was low with only 13 of 287 patients (4.5%). Histopathological and immunohistochemical analysis was performed on FFPE tissue specimens generated from the same biopsies, confirming the diagnosis of melanoma in all but four cases (1.4%), which showed sarcoidosis (n=1), as well as metastasis from colorectal cancer, lymphoma, and adenocarcinoma of unknown primary (each n=1). These patients did not receive study treatment and were excluded from PP analysis (see Figure 1). The remaining evaluable chemosensitivity test results revealed heterogenous sensitivity profiles to the chemotherapeutic combinations tested (Table 1). The ratio of chemosensitive to chemoresistant tumors was 0.34 and 0.39, respectively, in the two treatment arms. The rate of chemosensitive tumors was higher for both combinations containing treosulfan (TreoGem; TreoAraC) than for CisTax.

### Response to treatment

**Figure 2:**
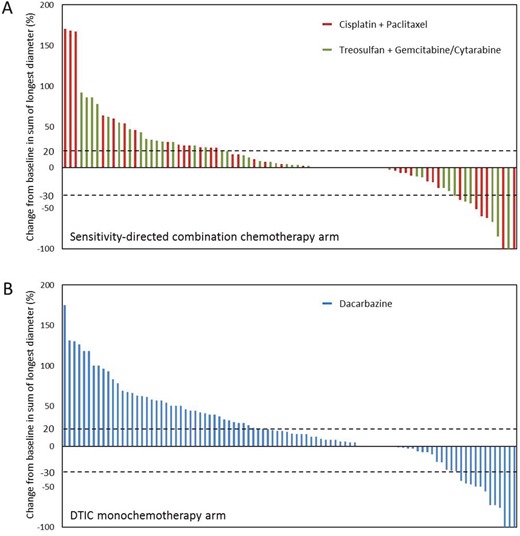
Waterfall plot depicting the best tumor response for each patient Data regarding the best tumor response are shown for 84 patients in the sensitivity-directed combination chemotherapy arm **(A)** and for 94 patients in the dacarbazine monochemotherapy arm **(B)**, who had undergone at least one tumor assessment after study treatment. Each bar represents data for an individual patient. Colors indicate the treatment regimen received by the patients. The percent change from baseline in the sum of the longest diameters of the target lesions defined at study entry is shown on the y axis. Negative values indicate tumor shrinkage; positive values indicate tumor growth. The pointed lines indicate changes in diameters corresponding to partial response (-30%) and progressive disease (+20%), respectively.

Assessed in the PP population, the objective response rate was not significantly different in chemosensitivity-directed combination chemotherapy as compared to dacarbazine monochemotherapy (10.7% vs 12.3%; p=0.90). The disease control rate showed a trend towards a better outcome in chemosensitivity-directed combination therapy (32.8% vs 23.0%); however, this difference did not reach statistical significance (p=0.088). Analysis of treatment response to the therapy regimens within the sensitivity-directed combination chemotherapy arm revealed no significant differences between the regimens containing treosulfan (TreoGem, TreoAraC) and the regimen containing paclitaxel (CisTax). With regard to *ex-vivo* chemosensitivity test results, chemosensitive tumors (BICSI≤100) revealed no significant differences in objective response and disease control as compared to chemoresistant tumors (BICSI>100). Detailed treatment response data are presented in Table 2, Figure 2 and Supplementary Table 1.

**Table 2:** Response and survival by treatment

	PP (n=244)			
	Standard chemotherapy (n=122)	Sensitivity-directed chemotherapy (n=122)		
	DTIC (n=122)	Total (n=122)	Treo + Gem/AraC (n=58)	Cis + Tax (n=64)
**Best response**				
CR	3 (2.5%)	3 (2.5%)	1 (1.7%)	2 (3.1%)
PR	12 (9.8%)	10 (8.2%)	5 (8.6%)	5 (7.8%)
SD	13 (10.7%)	27 (22.1%)	11 (19.0%)	16 (25.0%)
PD	91 (74.6%)	76 (62.3%)	40 (69.0%)	36 (56.3%)
Not evaluable	3 (2.5%)	6 (4.9%)	1 (1.7%)	5 (7.8%)
**Best response grouped**				
Objective response (CR+PR)	15 (12.3%)	13 (10.7%)	6 (10.3%)	7 (10.9%)
Disease control (CR+PR+SD)	28 (23.0%)	40 (32.8%)	17 (29.3%)	23 (35.9%)
**Survival times**				
Overall survival; median (95% CI)	9.0 (7.3; 11.6)	9.2 (8.0; 12.1)	9.0 (5.5;15.1)	9.8 (8.0;13.4)
Progression-free survival; median (95% CI)	2.3 (2.3; 2.5)	2.5 (2.3; 2.6)	2.3 (2.2;2.6)	2.6 (2.4;4.4)

Response and survival of the per-protocol (PP) population. Percentages are given per column, each representing a treatment group. Best response was defined as the best tumor response recorded from the start of treatment until removal of the patient from the trial. Survival was measured from the date of enrollment until the date of death or disease progression, respectively; if no such event occurred, the date of the last patient contact was used as endpoint. CI, confidence interval; CR, complete response; PR, partial response; SD, stable disease; PD, progressive disease.

### Survival analysis

**Figure 3:**
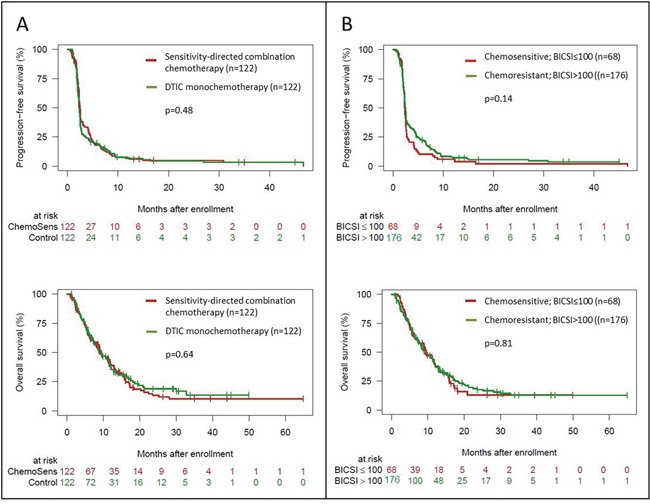
Kaplan Meier curves showing the probability of progression-free and overall survival of the per-protocol population (n=244) by treatment arm **(A)** and by *ex-vivo* determined tumor chemosensitivity. **(B)** Differences between groups were calculated using the log rank test. Censored observations are indicated by vertical bars.

At database lock in January 2015, 287 patients were enrolled and 244 patients were treated per-protocol. In the PP population, 179 patients died and the median follow-up time was 26.4 months. OS showed no significant difference between the chemosensitivity-directed combination therapy arm and the dacarbazine monochemotherapy arm (median 9.2 vs 9.0 months; p=0.64; Figure 3A). Accordingly, no significant difference was detected in PFS between the two arms (median 2.5 vs 2.3 months; p=0.48; Figure 3A). Survival time analysis of the two therapy regimens within the chemosensitivity-directed combination therapy arm revealed no significant differences in OS between the regimens containing treosulfan (TreoGem, TreoAraC) and the regimen containing paclitaxel (CisTax); p=0.62 (Supplementary Figure 1). PFS was significantly longer in patients receiving CisTax as compared to patients treated with a treosulfan-based regimen (median 2.6 vs 2.3 months; p=0.029); Supplementary Figure 1. With regard to *ex-vivo* chemosensitivity test results, patients with chemosensitive tumors (BICSI≤100) revealed no significantly different OS and PFS times than patients with chemoresistant tumors (BICSI>100); Figure 3B. Cox proportional hazards regression considering the factors therapy (combination chemotherapy vs dacarbazine) and *ex-vivo* chemosensitivity (BICSI≤100 vs BICSI>100) including the interaction of both factors showed that for OS and PFS the interaction term was not significantly different from 0 (p=0.5 for both), hence suggesting that the chemosensitivity test result BICSI was not predictive for survival. Without the interaction term, the model resulted in hazards ratios for OS of 1.08 (95%-confidence interval (CI) 0.80-1.45) for therapy and 1.03 (95%-CI 0.74-1.43) for *ex-vivo* chemosensitivity. For PFS, the hazard ratios were 0.91 (95%-CI 0.70-1.18) for therapy and 1.25 (95%-CI 0.94-1.67) for *ex-vivo* chemosensitivity. These results suggest, that the BICSI is not prognostic for either OS or PFS. Detailed survival data are presented in Table 2 and Supplementary Table 1.

### Prognostic factors

Patients with an elevated serum LDH at enrollment showed a poorer OS than patients with normal LDH levels (Supplementary Figure 2). This correlation was significant in the total PP population (p<0.0001) as well as in all subgroups (patients treated with chemosensitivity-directed combination therapy; patients treated with dacarbazine; patients whose tumors were tested chemosensitive; patients whose tumors were tested chemoresistant), without showing significant differences between those subgroups. Other parameters with a significant impact on PFS and OS were overall performance status (ECOG=0 vs ECOG≥1; p<0.0001 both; Supplementary Figure 2), M category (M1a vs M1b vs M1c; p=0.028 and p=0.003), and sum of longest diameters of target lesions (≤10 cm vs >10 cm; p=0.015 and 0.009). Again, these correlations were significant in the total PP population as well as in all subgroups mentioned above. The parameters age (p=0.65; Supplementary Figure 2), gender (p=0.068; Supplementary Figure 1), previous systemic therapy in stage III, and previous systemic therapy in stage IV (data not shown) revealed no significant impact on survival in the studied patients.

### Treatment-related toxicity

CTC grade 3 or 4 toxicities and the actions required by those are summarized in Table 3. These higher grade toxicities were experienced significantly more often by patients treated with a chemosensitivity-directed combination chemotherapy than by patients treated with dacarbazine monochemotherapy (40.2% vs 12.3%; p<0.0001). Using combination chemotherapy, the most frequently reported adverse events were neutropenia (17.2%), anemia (5.7%), pain (5.7%), increase in liver enzymes (4.9%), neuropathy (4.9%), and fatigue (3.3%). The most frequent adverse events observed in the dacarbazine arm were neutropenia and increase in liver enzymes (both 3.3%). Comparing the two regimens used in the chemosensitivity-directed combination therapy arm, CisTax appeared to have a lower frequency of severe toxicities than the combinations containing treosulfan (35.9% vs 44.8%; p=0.42). Drug hypersensitivity reactions, neuropathy, and fatigue were observed more frequently in patients treated with CisTax. Severe adverse events led to dose reductions more often in patients treated in the chemosensitivity-directed combination therapy arm than in patients treated with dacarbazine (18.0% vs 7.4%); however, treatment cycle delays were necessary equally frequent in both arms (both 12.3%). Treatment discontinuation became necessary in only two patients (1.6%) treated with dacarbazine (one patient presenting a grade 4 drug hypersensitivity reaction; one patient showing grade 4 renal failure and status epilepticus), but in 4 patients (3.3%) treated with combination chemotherapies. In the latter, all treatment discontinuations occurred in patients treated with CisTax (grade 3 neuropathy; grade 4 nausea, vomiting and fatigue; grade 3 diabetes; grade 4 drug hypersensitivity reaction), and none occurred in patients treated with treosulfan-containing regimens.

**Table 3:** Treatment-related severe toxicities

	PP (n=244)			
	Standard chemotherapy (n=122)	Sensitivity-directed chemotherapy (n=122)		
	DTIC (n=122)	Total (n=122)	Treo + Gem/AraC (n=58)	Cis + Tax (n=64)
**Treatment-related adverse events grade 3 or 4**				
**Any**	**15 (12.3%)**	**49 (40.2%)**	**26 (44.8%)**	**23 (35.9%)**
Anemia	2 (1.6%)	7 (5.7%)	4 (6.9%)	3 (4.7%)
Neutropenia	4 (3.3%)	21 (17.2%)	12 (9.8%)	9 (14.1%)
Thrombocytopenia	2 (1.6%)	3 (2.5%)	3 (2.5%)	-
Pyrexia	1 (<1%)	1 (<1%)	1 (1.7%)	-
Nausea/vomiting	-	1 (<1%)	-	1 (1.6%)
Decreased appetite/weight loss	-	2 (1.6%)	1 (1.7%)	1 (1.6%)
Digestive disorder	-	1 (<1%)	-	1 (1.6%)
Increase in liver enzymes	4 (3.3%)	6 (4.9%)	4 (6.9%)	2 (3.1%)
Increase in creatinine	1 (<1%)	-	-	-
Drug hypersensitivity reaction	1 (<1%)	3 (2.5%)	-	3 (4.7%)
Neuropathy	1 (<1%)	6 (4.9%)	2 (3.4%)	4 (6.3%)
Infection	1 (<1%)	-	-	-
Hemorrhagia	1 (<1%)	1 (<1%)	-	1 (1.6%)
Dyspnoe	-	2 (1.6%)	2 (3.4%)	-
Endocrinopathy	-	1 (<1%)	-	1 (1.6%)
Embolism/ischemic event	1 (<1%)	1 (<1%)	-	1 (1.6%)
Pain	1 (<1%)	7 (5.7%)	4 (6.9%)	3 (4.7%)
Fatigue	1 (<1%)	4 (3.3%)	-	4 (6.3%)
**Treatment-related adverse events leading to change in therapy schedule**				
Dose reduction	9 (7.4%)	22 (18.0%)	9 (15.5%)	13 (20.3%)
Cycle delay	15 (12.3%)	15 (12.3%)	5 (8.6%)	10 (15.6%)
Treatment discontinuation	2 (1.6%)	4 (3.3%)	-	4 (6.3%)

Toxicity was classified and graded according to CTC 3.0 (http://ctep.cancer.gov/reporting/ctc.html). Data represent the worst CTC grade experienced by each patient.

## DISCUSSION

The present trial demonstrates pre-therapeutic chemosensitivity testing of freshly obtained tumor tissue samples using an *ex-vivo* ATP-based chemosensitivity assay as a feasible method in metastatic melanoma, even in a multicenter setting using one central test laboratory. Indeed, in only 4.5% of patients the test assay revealed non-evaluable results, mainly due to low numbers of viable tumor cells obtained from the respective tissue biopsies. A careful histopathological review of all biopsy materials subjected to chemosensitivity testing proved to be highly reasonable, since by this means a significant number of samples (1.4%) were disclosed as to be derived from other malignancies than melanoma.

Living tumor cells derived from fresh tumor biopsies revealed heterogenous *ex-vivo* chemosensitivity profiles, with about one third of patients categorized as chemosensitive, and two thirds categorized as chemoresistant according to an arbitrary cut-off defined as a BICSI of 100, which showed to be the best discriminator of chemosensitivity within the preceding phase-2 trial. However, patients whose tumors were categorized as chemosensitive by this means presented no significant differences in objective response (CR+PR) and disease control (CR+PR+SD) as compared to patients whose tumors were categorized as chemoresistant. Also, survival in terms of PFS and OS showed no significant differences between patients with chemosensitive and patients with chemoresistant tumors. Thus, the *ex-vivo* chemosensitivity test result BICSI was neither prognostic nor predictive in the patient cohort investigated in the present study.

Testing for superiority of the experimental treatment arm, chemosensitivity-directed combination chemotherapy, against the control arm, dacarbazine monochemotherapy, revealed no significant difference in objective response (CR+PR). Disease control (CR+PR+SD) showed a trend towards a favorable outcome in the experimental arm, not reaching statistical significance. However, survival neither for OS, which was the primary endpoint of this trial, nor for PFS revealed significant differences between the two treatment arms.

The experimental arm of this trial included different chemotherapy regimens, which proved as most efficacious in the previous phase-2 study: a platin-based regimen (CisTax) and two treosulfan-based regimens (TreoGem, TreoAraC), which never have been tested head-to-head within a prospective clinical study before. The present trial revealed no significant differences in therapy response between either regimens. With regard to survival, PFS was significantly prolonged in patients receiving CisTax as compared to patients treated with a regimen containing treosulfan. However, this survival benefit did not translate into OS, which showed no significant differences between regimens. With regard to response, the objective response rate was not significantly different between regimens. Still, the disease control rate tended to be higher in patients receiving CisTax.

Analysis of therapy-related toxicity clearly showed that severe toxicities of CTC grade 3 or 4 were more than three times more often observed in patients treated with combination chemotherapy regimens than in patients treated with dacarbazine monochemotherapy (40.2% versus 12.3%). This difference was highly significant with p<0.0001. With this high frequency of severe adverse events, the combination chemotherapy regimens tested in the present trial are close to the range of those observed under combination immune checkpoint inhibition, with e.g. grade 3 or 4 events under ipilimumab + nivolumab of about 50% {Postow, 2015 [14] 1250/id;Larkin, 2015 [15] 1256/id}. The most common severe adverse events observed in the present trial were neutropenia, anemia, pain, hepatotoxicity, neuropathy, and fatigue. Interestingly, severe nephrotoxicity, which is known to be more frequent in cisplatin-based regimens as compared to carboplatin-based regimens, was not reported in any combination chemotherapy regimen of the present trial.

The striking difference in the frequency of severe toxicity between combination and monochemotherapy observed in the present trial is of particular interest for the current situation of clinical care for metastatic melanoma patients. Today, chemotherapy often is a second-line or higher treatment option in patients refractory or not suitable for kinase inhibitor or checkpoint inhibitor therapies. These patients frequently are in reduced health condition, and further deterioration of their health status by therapeutic interventions should be avoided. The results of the present trial indicate, that with no significant benefit in response and survival, but showing significantly higher rates of severe toxicities, combination therapies with CisTax or TreoGem/AraC should not be the chemotherapy regimens of the first choice. Instead, dacarbazine monochemotherapy should be used leading to comparable therapy response and survival results with a three times lower frequency of severe toxicities.

It should be noted, that because of the substantial shift in the treatment algorithms on advanced melanoma recruitment into this trial had to be stopped early, before the planned number of 360 patients could be reached. Thus, only 274 patients were enrolled with intention-to-treat, and the study was not adequately powered with reard to its primary endpoint OS. However, the OS data collected from the 244 patients who were evaluable per-protocol revealed this highly overlapping survival curves between all respective groups (chemosensitivity-directed combination chemotherapy, dacarbazine monochemotherapy, chemosensitive tumors, chemoresistant tumors), that it seems highly unlikely that a recruitment of the full intendend number of patients would have revealed a difference.

An important question remains for the reasons leading to the failure of the present trial to meet its goals, in particular with regard to the preceding phase-2 trial providing promising results. The phase-2 trial included a much smaller number of patients, with only 53 patients evaluable per-protocol. In that patient cohort, response rates were higher (36% in chemosensitive vs 16% in chemoresistant patients) than in the present trial (10% vs 12%). Also, OS was longer in these patient groups (14.6 vs 7.4 months) as compared to the current trial (9.5 vs 9.0 months). Thus, the promising data observed in the phase-2 trial could be related to patient selection and bias due to low patient numbers and a considerably lower number of study centers. It is important to note, that the introduction of new therapeutic strategies for metastatic melanoma, such as BRAF/MEK inhibition and immune checkpoint blockade, is unlikely to be the reason for the failure of the present trial. Less than 10% of patients of either treatment arms received one of these, and if so, mostly after study treatment, with frequencies balanced between arms. Thus, we assume no interference between the use of new treatment strategies and the results of the present study.

Taken together, the aim of this trial to demonstrate a superiority of an individualized chemosensitivity-directed combination chemotherapy against the standard dacarbazine monochemotherapy was not reached. The second aim of this trial, to demonstrate a prognostic value of the *ex-vivo* chemosensitivity test result BICSI, was also not achieved. Based on our results, the ATP-based *ex-vivo* chemosensitivity assay used in the present trial cannot be recommended for the clinical use in melanoma patients outside of study protocols, as previously recommended by the ASCO Working Group on Chemotherapy Sensitivity and Resistance Assays [16]. In consideration of the significantly higher frequency of severe adverse events observed in the present trial under chemotherapy with CisTax, TreoGem, and TreoAraC, dacarbazine should be preferred over these combination regimens if chemotherapy is considered as an option in the treatment of metastatic melanoma patients.

## PATIENTS AND METHODS

### Study design

The primary endpoint of this multicenter randomized open-label prospective phase-3 trial (clincalTrials.gov: NCT00779714) was overall survival (OS), secondary endpoints were best response, progression-free survival (PFS) and toxicity. All endpoints were evaluated on intention-to-treat (ITT) and per-protocol (PP) basis, besides toxicity which was only evaluated in patients who received at least one course of treatment. The trial was aimed to test simultaneously for (i) the prognostic value of the best individual chemosensitivity index (BICSI) as measured by an *ex-vivo* ATP-based chemosensitivity assay, i.e. comparing the chemosensitive (BICSI≤100) with the chemoresistant (BICSI>100) patients, and (ii) for superiority of an individualized chemosensitivity-directed combination chemotherapy against dacarbazine monochemotherapy. Based on the results of the preceding phase-2 trial [13], the study was designed to enroll 360 patients in order to detect a 30% difference in OS between individualized chemosensitivity-directed combination chemotherapy and dacarbazine monochemotherapy (expected OS: 12 vs 8 months) as well as to detect a 30% difference in OS between chemosensitive and chemoresistant patients (expected OS: 12 vs 8 months, distributed with a 2:3 ratio), both with a statistical power of 90%, and alpha=0.025 in a one-sided log rank test, limiting the overall false positive rate to 5%. Besides these primary aims, the study was intended to perform subgroup analyses on a potential difference between chemosensitivity-directed chemotherapy and dacarbazine monochemotherapy in chemosensitive or chemoresistant patients only. For this pupose, the marker-by-treatment interaction study design [17] was chosen, which allows a cross-over survival analysis of patients treated in different therapy arms.

### Patient population

Patients with histologically confirmed metastatic melanoma and surgically unresectable distant metastases were enrolled in accordance with the following major eligibility criteria: stage IV disease following AJCC criteria [18]; no previous systemic chemotherapy in stage IV; at least one measurable target lesion following the response evaluation criteria in solid tumors (RECIST) [19]; access to a fresh biopsy of ~1 cm^3^ from a metastatic lesion for *ex-vivo* chemosensitivity testing; ECOG overall performance status of 0 or 1; willing and physically able to receive polychemotherapy; age≥18 years; adequate bone marrow function (hemoglobin≥9 g/dl, absolute neutrophil count≥1500/μl, platelets≥100.000/μl); and satisfactory hepatic and renal functions. All types of metastatic sites were considered eligible besides metastases to the brain; former history of brain metastases, which had been treated successfully and are no longer visible in CT/MRI was allowed. Primary cutaneous or mucosal melanomas, as well as melanomas of unknown primary were eligible; primary ocular melanomas were excluded. The study protocol was approved by the central Institutional Review Board (Medizinische Ethikkommission der Universität Würzburg 123/08) as well as by the respective Institutional Review Boards of all participating centers. A written informed consent had to be signed by all patients prior to enrollment.

### Chemosensitivity assay

After patient registration, an excisional biopsy of a metastatic lesion was taken and shipped to the central test laboratory within 24 hours. There, connective and fatty tissues were removed, and ~1 cm^3^ of tumor tissue was subjected to chemosensitivity testing. The remaining tumor tissue was used partly for cryopreservation, and partly to generate formalin-fixed and paraffin-embedded (FFPE) samples for immunohistochemical confirmation of melanoma diagnosis. Chemosensitivity testing was performed using a non-clonogenic ATP-based luminescence assay (ATP-TCA, DCS Innovative Diagnostic Systems, Hamburg, Germany) [12, 13]. Briefly, the tissue samples were minced and thereafter enzymatically dissociated. The obtained single cell suspensions were depleted of red blood cells and debris by Ficoll-Hypaque density gradient centrifugation and thereafter assessed for tumor cell count and viability by trypan blue dye exclusion. Minimum tumor cell viability was defined as 25%, otherwise the assay was considered inevaluable. The cell suspensions were given into polypropylene round-bottom 96-well plates (2×10^4^ cells/well) with or without different chemotherapeutic agents at six different dilutions (6.25, 12.5, 25, 50, 100, 200) of the individual test drug concentrations (TDC), each tested in triplicates. The drugs and TDCs used were 20 μg/ml dacarbazine, 3.8 μg/ml cisplatin, 13.6 μg/ml paclitaxel, 12.5 μg/ml gemcitabine, 2.4 μg/ml cytarabine, and 20 μg/ml treosulfan. After seven days of incubation at 37°C, 5% CO_2_ and 100% humidity, the cells were lysed and their ATP content was quantified by a luciferin-luciferase luminescence reaction using a microplate luminometer (Berthold Detection Systems, Pforzheim, Germany). Cell suspensions incubated without cytotoxic drugs were used as reference for 100% tumor cell viability.

### Best individual chemosensitivity index (BICSI) and randomization

Individual chemosensitivity indices ranging from 0 to 600 for each test drug or drug combination were calculated as described before [12, 13]. Thus, a sensitivity index of 600 indicates full cell viability/minimal drug sensitivity, whereas a sensitivity index of 0 reflects complete cell death/maximal drug sensitivity. The lowest individual chemosensitivity index resulting from *ex-vivo* drug testing, corresponding to the highest individual chemosensitivity, was calculated for each patient and defined as the best individual chemosensitivity index (BICSI). Based on the results of the preceding phase-2 trial [13], patients whose tumors were tested with a BICSI≤100 were considered chemosensitive, whereas patients whose tumors showed a BICSI>100 were considered chemoresistant. Patients with evaluable chemosensitivity test results were randomized based on the strata *ex-vivo* chemosensitivity (BICSI≤100; BICSI>100), M category (M1a/b; M1c), and ECOG overall performance status (0; 1) between the two treatment arms (chemosensitivity-directed combination chemotherapy; dacarbazine monochemotherapy) in a 1:1 ratio using separate block randomization for each stratum.

### Sensitivity-directed chemotherapy

Patients randomized into the chemosensitivity-directed combination chemotherapy arm received the drug combinations showing the highest *ex-vivo* sensitivity out of three combinations tested (cisplatin+paclitaxel, CisTax; treosulfan+gemcitabine, TreoGem; treosulfan+cytarabine, TreoAraC). This means, in this study arm patients were treated with the drug combination resulting in the lowest *ex-vivo* tumor cell viability, corresponding to the highest *ex-vivo* tumor cell kill, irrespective of the *ex-vivo* categorization of the patient's tumor as chemosensitive or chemoresistant. The therapy regimens used were CisTax: paclitaxel 200 mg/m^2^ i.v. for 180 min, cisplatin 50 mg/m^2^ i.v. for 60 min, intermitted by a 1 hour interval, repeated every 21 days; TreoGem: gemcitabine 1250 mg/m^2^ i.v. for 30 min, treosulfan 3500 mg/m^2^ i.v. for 30 min, intermitted by a 3 hours interval, repeated every 21 days; TreoAraC: cytarabine 100 mg/m2 i.v. for 24 h, days 1-3, treosulfan 3500 mg/m2 i.v. for 30 min, day 2, repeated every 21 days. Patients randomized into the dacarbazine monochemotherapy arm received dacarbazine 1000 mg/m^2^ i.v. for 30 min, repeated every 21 days. Treatment was continued until disease progression or intolerable side effects; treatment beyond progression was allowed at the discretion of the investigator. Recommended concomitant medications were serotonin antagonists in all regimens, and corticosteroids as well as histamine antagonists in regimens containing paclitaxel. Toxicity was evaluated in all patients who received study treatment using common toxicity criteria (CTC) 3.0 (http://ctep.cancer.gov/reporting/ctc.html).

### Response and survival assessment

Patients who completed at least one cycle of chemotherapy were considered evaluable for response. Tumor response was assessed by CT and/or MRI imaging in 8-weeks intervals and evaluated according to RECIST [19]. Complete (CR) and partial (PR) responses were combined as objective response (OR). All ORs had to be confirmed by repeated CT or MRI scans. Patients who died from melanoma rapidly after onset of study treatment, so that no assessment of tumor response could be done, were considered as progressive disease (PD) [19]. Best response was defined as the best response recorded from the start of treatment until disease progression; best responses of SD or better (CR+PR+SD) were considered as disease control. OS and PFS were measured from therapy onset until death or disease progression, respectively. If no such event occurred, the date of the last patient contact was used as endpoint of survival assessment (censored observation).

### Statistical analysis

The database was locked in January 2015. Survival endpoints were calculated using the Kaplan-Meier method for censored failure time data. The two-sided log rank test was used for comparison of survival between groups. Cox proportional hazards regression was used to estimate hazard ratios. Chi-square test was used to compare tumor response rates and toxicities between groups. All analyses were carried out using the software R 3.3.1.

## SUPPLEMENTARY MATERIALS FIGURES AND TABLE


