# Supplementary material for: Chemosensitivity-directed therapy compared to dacarbazine in chemo-naive advanced metastatic melanoma: a multicenter randomized phase-3 DeCOG trial
Source: Oncotarget. 2017 Jun 27;8(44):76029–43. doi: 10.18632/oncotarget.18635 (PMC5652683; doi:10.18632/oncotarget.18635)
Supplement: Supplementary file 1 [file oncotarget-08-76029-s001.pdf]

Chemosensitivity-directed therapy compared to dacarbazine in chemo-naive advanced metastatic melanoma: a multicenter randomized phase-3 DeCOG trial

SUPPLEMENTARY FIGURES AND TABLE

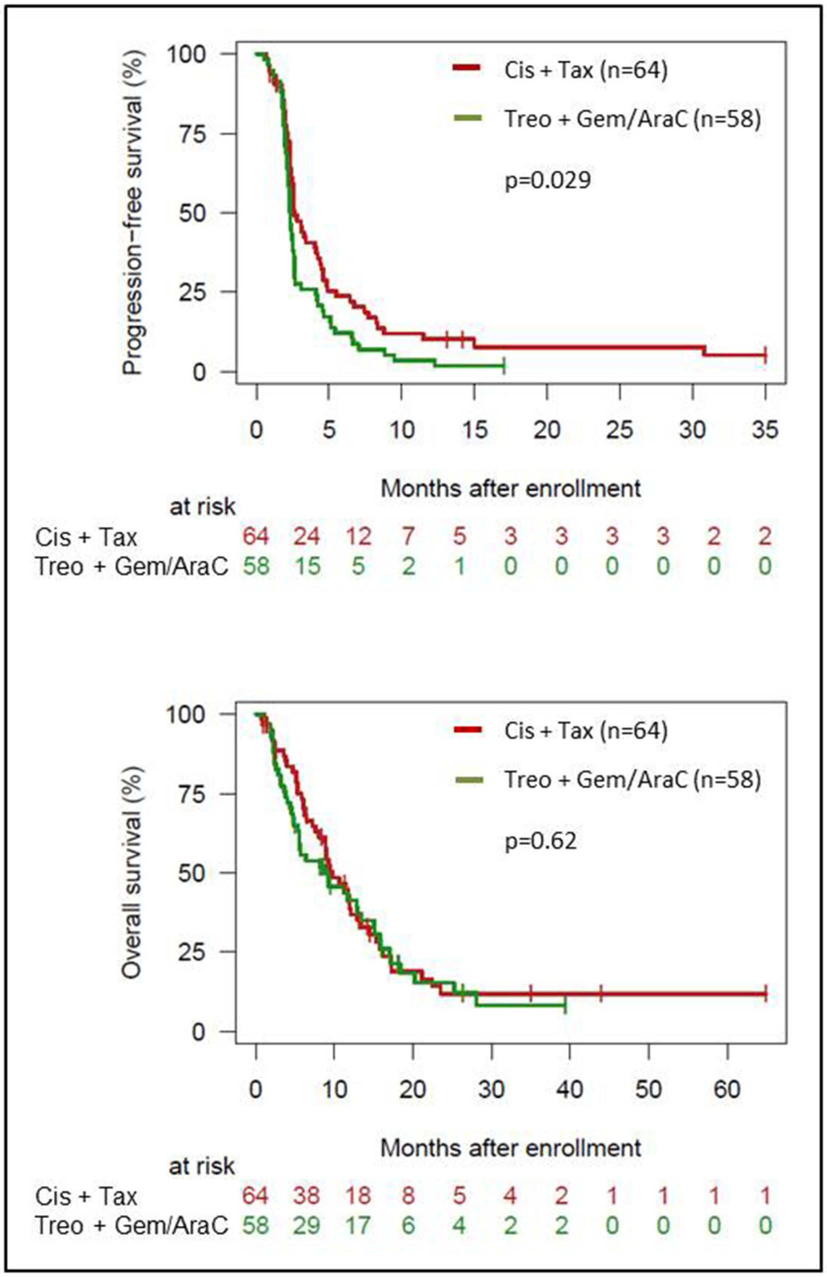

Supplementary Figure 1: Kaplan Meier curves showing the probability of progression-free and overall survival of the per-protocol population treated in the sensitivity-directed combination chemotherapy arm (n=122) by different treatment regimens, cisplatin + paclitaxel (Cis + Tax) versus treosulfan + gemcitabine/cytarabine (Treo + Gem/AraC). Differences between groups were calculated using the log rank test. Censored observations are indicated by vertical bars.

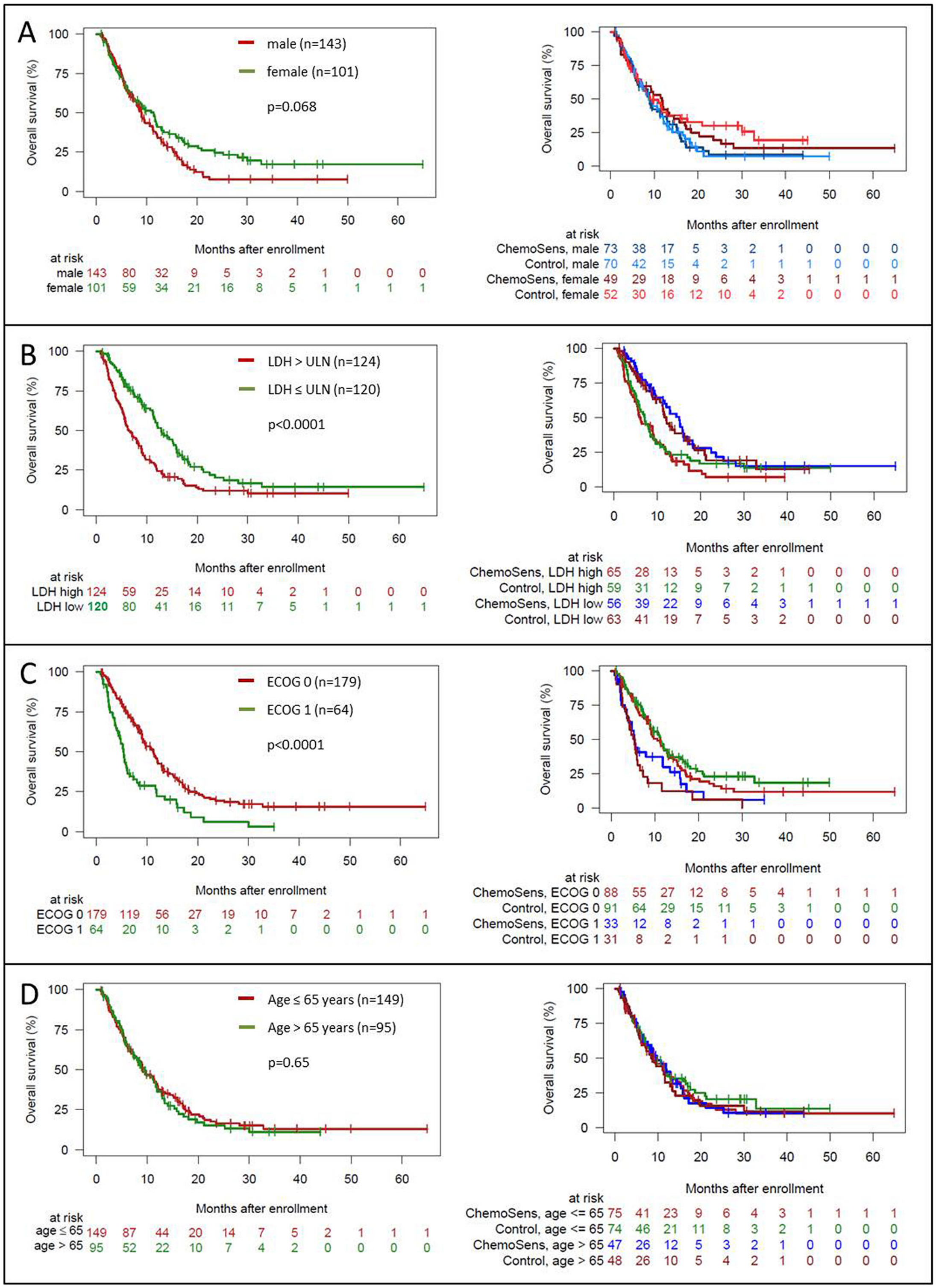

Supplementary Figure 2: Kaplan Meier curves showing the probability of overall survival of the per-protocol population (n=244) by clinical parameters at study enrollment (left panels) and additionally by treatment arm (right panels). Clinical parameters are **(A)** gender, **(B)** serum lactate dehydrogenase, **(C)** overall performance status categorized according to ECOG criteria, and **(D)** age. Differences between groups were calculated using the log rank test. Censored observations are indicated by vertical bars. Abbreviations: ChemoSens, sensitivity-directed combination chemotherapy arm; control, dacarbazine monochemotherapy arm; LDH, lactate dehydrogenase; ULN, upper limit of normal; ECOG, Eastern Cooperative Oncology Group.

Supplementary Table 1: Response and survival by ex-vivo chemosensitivity

	PP (n=244)	
	**Chemosensitive (BICSI≤100) n=68**	**Chemoresistant (BICSI>100) n=176**
**Best response**		
CR	2 (2.9%)	4 (2.3%)
PR	5 (7.4%)	17 (9.7%)
SD	9 (13.2%)	31 (17.6%)
PD	52 (76.5%)	115 (65.3%)
Not evaluable	0 (0.0%)	9 (5.1%)
**Best response grouped**		
Objective response (CR+PR)	7 (10.3%)	21 (11.9%)
Disease control (CR+PR+SD)	16 (23.5%)	52 (29.5%)
**Survival times**		
Overall survival; median (95% CI)	9.5 (7.3; 13.0)	9.0 (8.0; 11.6)
Progression-free survival; median (95% CI)	2.3 (2.3; 2.6)	2.5 (2.3; 2.6)

Response and survival of the per-protocol (PP) population. Percentages are given separately per column, representing patients whose tumor tissue was categorized as chemosensitive or chemoresistant by an ex-vivo ATP-based chemosensitivity assay. Best response was defined as the best tumor response recorded from the start of treatment until removal of the patient from the trial. Survival was measured from the date of enrollment until the date of death or disease progression, respectively; if no such event occurred, the date of the last patient contact was used as endpoint. CI, confidence interval; CR, complete response; PR, partial response; SD, stable disease; PD, progressive disease.
